# Palmitic acid induces lipid droplet accumulation and senescence in nucleus pulposus cells via ER-stress pathway

**DOI:** 10.1038/s42003-024-06248-9

**Published:** 2024-05-07

**Authors:** Xi Chen, Kun Chen, Jun Hu, Yijun Dong, Menglong Zheng, Jiang Jiang, Qingsong Hu, Wenzhi Zhang

**Affiliations:** 1 Department of Orthopedics, The First Affiliated Hospital of USTC, Division of Life Sciences and Medicine, University of Science and Technology of China, Hefei, 230001 China; 2 Department of Radiology, The First Affiliated Hospital of USTC, Division of Life Sciences and Medicine, University of Science and Technology of China, Hefei, 230001 China; 3 Department of Hepatobiliary Surgery, Anhui Province Key Laboratory of Hepatopancreatobiliary Surgery, The First Affiliated Hospital of USTC, Division of Life Sciences and Medicine, University of Science and Technology of China, Hefei, 230001 China

**Keywords:** Cell biology, Diseases

## Abstract

Intervertebral disc degeneration (IDD) is a highly prevalent musculoskeletal disorder affecting millions of adults worldwide, but a poor understanding of its pathogenesis has limited the effectiveness of therapy. In the current study, we integrated untargeted LC/MS metabolomics and magnetic resonance spectroscopy data to investigate metabolic profile alterations during IDD. Combined with validation via a large-cohort analysis, we found excessive lipid droplet accumulation in the nucleus pulposus cells of advanced-stage IDD samples. We also found abnormal palmitic acid (PA) accumulation in IDD nucleus pulposus cells, and PA exposure resulted in lipid droplet accumulation and cell senescence in an endoplasmic reticulum stress-dependent manner. Complementary transcriptome and proteome profiles enabled us to identify solute carrier transporter (SLC) 43A3 involvement in the regulation of the intracellular PA level. SLC43A3 was expressed at low levels and negatively correlated with intracellular lipid content in IDD nucleus pulposus cells. Overexpression of SLC43A3 significantly alleviated PA-induced endoplasmic reticulum stress, lipid droplet accumulation and cell senescence by inhibiting PA uptake. This work provides novel integration analysis-based insight into the metabolic profile alterations in IDD and further reveals new therapeutic targets for IDD treatment.

## Abstract (web summary)

Lipid droplet and palmitic acid accumulation in intervertebral disc degeneration nucleus pulposus cells drive endoplasmic reticulum stress-dependent cellular senescence.

## Introduction

Intervertebral disc degeneration (IDD) is the most common cause of low back pain in adults, and it can lead to patient disability and a large socioeconomic burden worldwide^1^. The intervertebral disc is an avascular organ consisting of the inner gel-like nucleus pulposus (NP), the outer annulus fibrosus, and superior and inferior cartilage endplates^2^. According to previous mechanistic studies, IDD is a cell-mediated process originating from central disc NP tissues and follows a programmed degeneration process^3^. A decrease in the number of cells in NP tissues is an obvious and important feature during IDD, during which residual NP cells are in a low proliferative state^4^. As an age-related musculoskeletal disorder, NP cell senescence is inevitably involved in the initiation and progression of IDD; however, a subset of individuals seem to be more susceptible to premature cell senescence and accelerated disc degeneration in an age-independent manner^5,6^. Under the actions of many pathological events, such as oxidative stress, endoplasmic reticulum (ER) stress, the inflammatory response or autophagy defects, the irregular activities of NP cells accelerate the initiation and development of premature cell senescence, also known as stress-induced premature senescence^7–9^. In both human patients and mouse models, NP cell senescence has been correlated with degenerative IDD phenotypes^6,10^. However, despite the known relationship between NP cell senescence and IDD, the mechanisms associated with the stress-induced premature senescence of NP cells are still not well characterized.

Degenerated intervertebral discs are commonly accompanied by stress-inducing conditions such as an acidic pH, low nutrient and high reactive oxygen species levels, inflammation, and lipotoxic metabolite accumulation^11^. The ER is a dynamic intracellular organelle that supports many functions, including protein folding and maturation, lipid synthesis, and calcium storage and release, all of which are essential for cellular homeostasis and the cellular response to the aforementioned stress conditions^12^. These pathological insults disrupt ER homeostasis and activate the unfolded protein response (UPR), an ER-stress-coping response that maintains ER function and cell survival^13^. However, excessive and prolonged ER stress can trigger premature cell senescence via the release of Ca2+ or the activation of a series of signal transduction pathways^13–15^. As experimentally validated in human IDD samples, increased cell senescence concomitant with increased expression of ER-stress markers has been previously observed^16^. The microenvironment of NP cells is regulated by the dynamic interplay of multiple factors, such as signaling molecules and pathways and specific metabolites inside and outside NP cells^5,11^. Hence, a thorough understanding of the molecular changes at multiple levels during IDD is essential for clarifying the complex relationships among stress conditions, ER stress, and NP cell senescence.

The applications of complementary multiomics approaches facilitate the characterization of the pathogenesis and identification of the mechanisms underlying diseases at multiple levels, and these data are interpreted to gain an understanding of complex global regulatory networks^17,18^. Through degeneration, NP cell signaling triggers metabolic or transcriptional regulatory networks, including senescence pathways^5^. All these alterations exert a substantial impact on biosynthesis, lead to the accumulation of various metabolites, and strongly influence stress conditions. The metabolomic analysis is a powerful approach for discovering metabolic profile alterations and elucidating the underlying mechanisms that are disrupted under stress conditions^19,20^. Moreover, metabolomic analysis data are increasingly being integrated with other omics data, such as proteomic and transcriptomic information, to increase the interpretability of metabolomic profiles^21^. The integration of multiomics data has helped to elucidate mechanisms that drive diseases and to discover putative molecules and therapeutic targets^19^. Recently, a multiomics approach was applied in osteoarthritis, prostate cancer, and nonalcoholic fatty liver disease and successfully revealed molecular alterations and potential therapeutic targets^17,19,22^. However, relatively little is known about the metabolic alterations and up- and downstream regulatory mechanisms as NP cells undergo senescence.

In this study, we performed a metabolomics analysis of human IDD samples aiming to identify key metabolic alterations unique to senescent NP cells. We then performed a transcriptomic and proteomic analysis to identify differentially expressed genes, which are thought to reflect senescent NP cell-specific metabolic changes. The corresponding metabolic and genetic alterations were validated with data from a separate and larger cohort of IDD patients. Finally, we integrated pathological phenotype information with transcriptomic, proteomic, and metabolomic data to perform an in-depth analysis of disrupted pathways at both the transcript, protein, and metabolite levels and to present a detailed explanation of the complex regulatory relationships among stress conditions, ER stress, and NP cell senescence.

## Results

### ER stress was detected during IDD

**Fig. 1:**
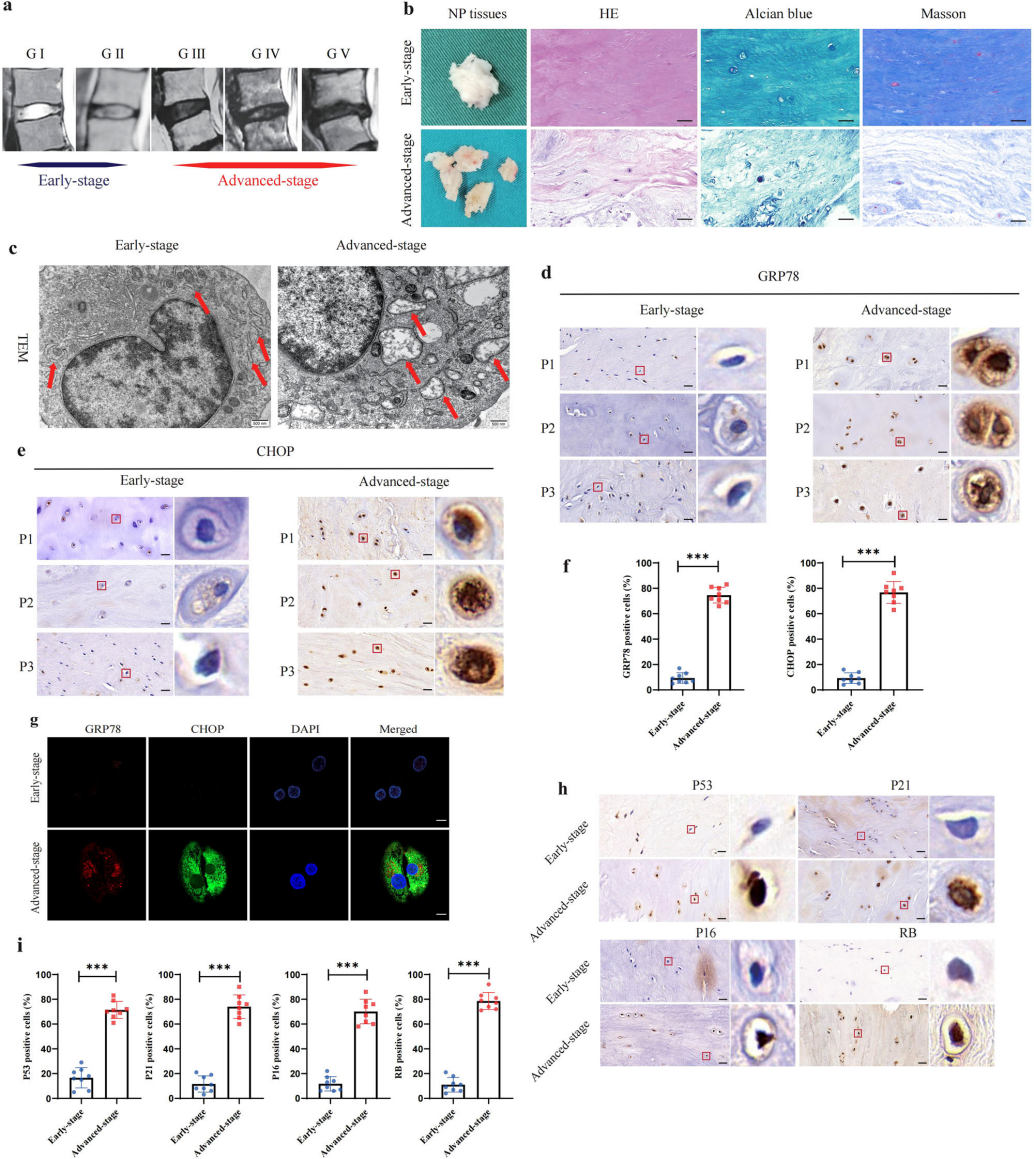
ER stress was detected during IDD. **a** Representative human T2-weighted MR images of intervertebral disks from grades I to V according to the Pfirrmann system. The intervertebral disks graded at I–II were defined as early-stage, and III–V were defined as advanced-stage. **b** The histological degenerative degrees of early- and advanced-stage NP tissues were measured by HE, Alcian blue, and Masson staining. Scale bar: 50 μm. **c** Transmission electron microscopy(TEM) observation revealed swollen and distention of the ER in degenerated NP cells. Scale bar: 2 μm. **d**–**f** Immunohistochemistry (IHC) staining of GRP78 and CHOP in human NP tissues. Scale bar: 50 μm. The statistical result of the positive cell rate was shown in a chart, *n* = 8. ***P < 0.001. **g** Immunofluorescence (IF) staining of GRP78 and CHOP in NP cells. Scale bar: 10 μm. **h**, **i** IHC staining of P53, P21, P16, and RB in human NP tissues. Scale bar: 50 μm. The result was quantified and shown in a chart, *n* = 8. ***P < 0.001.

To better understand the relationships among ER stress, NP cell senescence, and IDD, we collected NP tissues from patients who received lumbar discectomy due to low back pain and/or radiculopathy. The degree of IDD degenerated was graded on the basis of T2-weighted MR images, in which Grades I–II were collectively considered to indicate early-stage degeneration and Grades III–V were collectively considered to indicate advanced-stage degeneration (Fig. 1a). A total of 16 interverbal disks from 16 different patients were collected, and 4 samples were identified with Grade I degeneration, 4 samples were identified with Grade II degeneration, 2 samples were identified with Grade III degeneration, 3 samples were identified with Grade IV degeneration, and 3 samples were identified with Grade V degeneration. Representative histological images of early- and advanced-stage NP tissues after HE, Alcian blue, and Masson staining are shown in Fig. 1b. To identify alterations in cell ultrastructure, transmission electron microscopy (TEM) was performed to obtain clearer images of the cells. TEM revealed severe swelling and distention of the ER in degenerated NP cells, indicating ER stress (Fig. 1c). Protein markers for ER stress, namely, GRP78 and CHOP, were measured via immunohistochemistry (IHC) and immunofluorescence (IF) assays. The proportion of GRP78-positive and CHOP-positive cells was significantly higher in the advanced stage than in the early-stage samples (Fig. 1d–f). The IF results also showed that the expression levels of GRP78 and CHOP were higher in the advanced-stage samples than in the early-stage samples (Fig. 1g). In addition, the IHC analysis showed that the levels of P53, P21, P16, and RB expression were higher in the advanced-stage samples than in the early-stage samples (Fig. 1h–i). In vitro, TM, PA, and 2-DG induce ER stress and cell senescence after 72 h of treatment; Conversely, when TM, PA, or 2-DG are co-cultured with ER-stress inhibitor 4-PBA at the same time for 72 h, ER stress is suppressed and the levels of cell senescence are also alleviated (Supplementary Fig. 1a–e). These findings suggest a potential association between the activation of ER stress and NP cell senescence in IDD.

### In vivo magnetic resonance spectroscopy (MRS) analysis revealed lipid accumulation in IDD samples

**Fig. 2:**
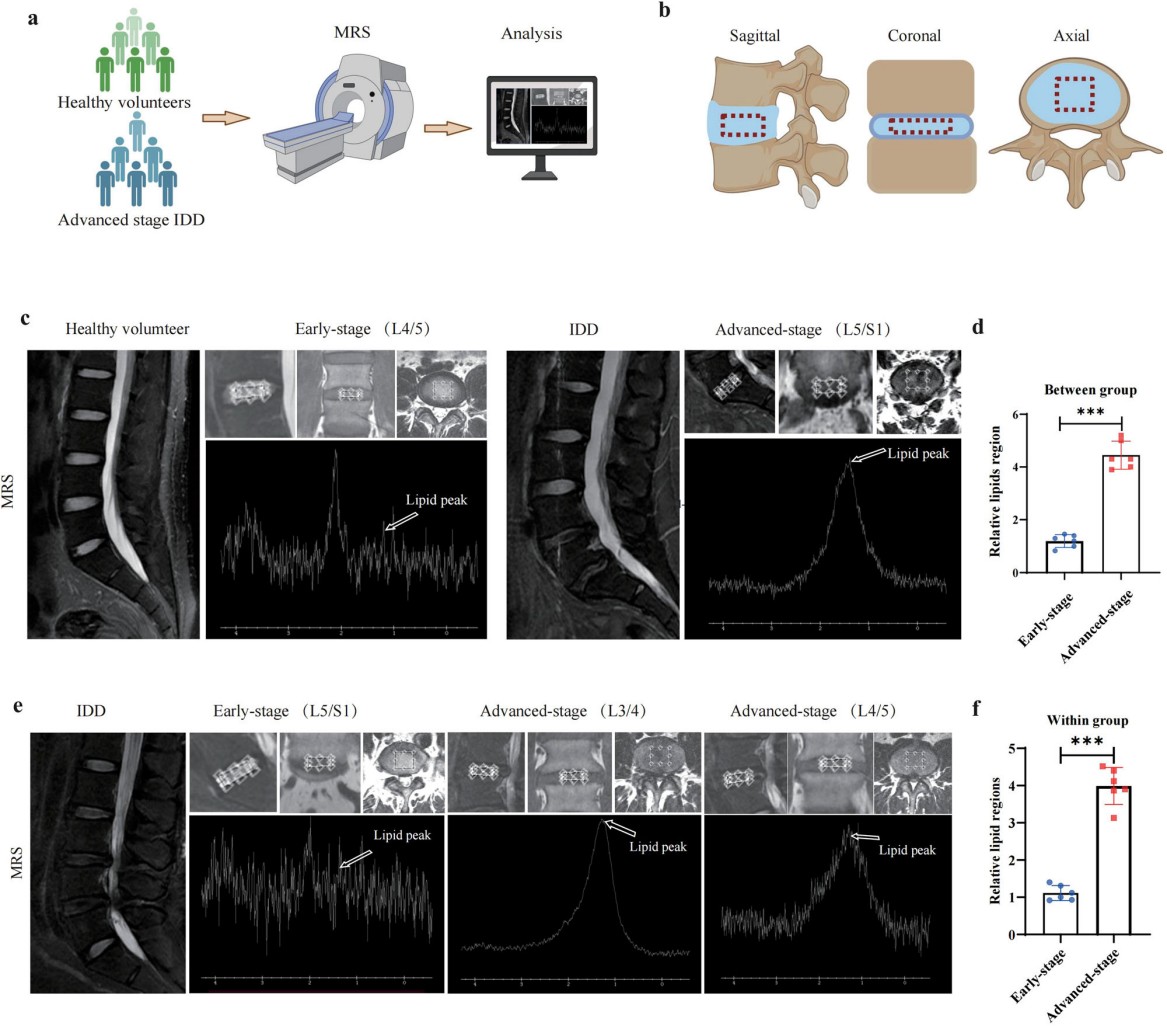
MRS analysis identifies lipid accumulation in IDD. **a** Workflow of MRS analysis. **b** Illustration of 3-plane voxel prescription for MRS analysis (left: mid-sagittal, center: coronal; right: axial). **c** MRS analysis for healthy volunteer and IDD patients, respectively. The spectrum showed a higher peak at 1.3 ppm, indicating lipid accumulation in IDD. **d** Statistical result of the relative area under the lipid peak between early- and advanced-stage groups. *n* = 6. ***P < 0.001. **e** MRS analysis of disks within different degenerated segments for each patient, and the spectrum demonstrated a higher lipid peak for degenerated disc (advanced-stage) and a relatively lower lipid peak for healthy disc (early-stage). **f** Statistical result of the relative area under the lipid peak within-group comparisons. *n* = 6. ***P < 0.001.

MRS is a noninvasive and nondiscriminating technique, making it an ideal tool for in vivo metabolic profiling of any organism. In the current study, we performed an NP tissue metabolic analysis using the MRS approach. A chart of the study workflow is shown in Fig. 2a (created with BioRender.com). The enrolled subjects consisted of 6 healthy volunteers and 6 IDD patients. The regions of interest are shown in the red dashed box in the scanned image (Fig. 2, created with BioRender.com). Compared with healthy volunteers, the MRS analysis of IDD patients revealed a higher peak at approximately 1.3 ppm, a site that represents lipids; the area under the lipid peak of the IDD patients was significantly higher than that of the healthy volunteers, indicating lipid accumulation (Fig. 2c, d). To reduce interpatient variability, we performed an intrapatient comparison between pairs of disks with different degenerated severities, and the MRS intrapatient analysis led to results to the initial findings (Fig. 2e, f). These findings suggest abnormal lipid accumulation in IDD.

### Metabolomic sequencing led to the identification of abnormal lipid droplet accumulation in IDD

**Fig. 3:**
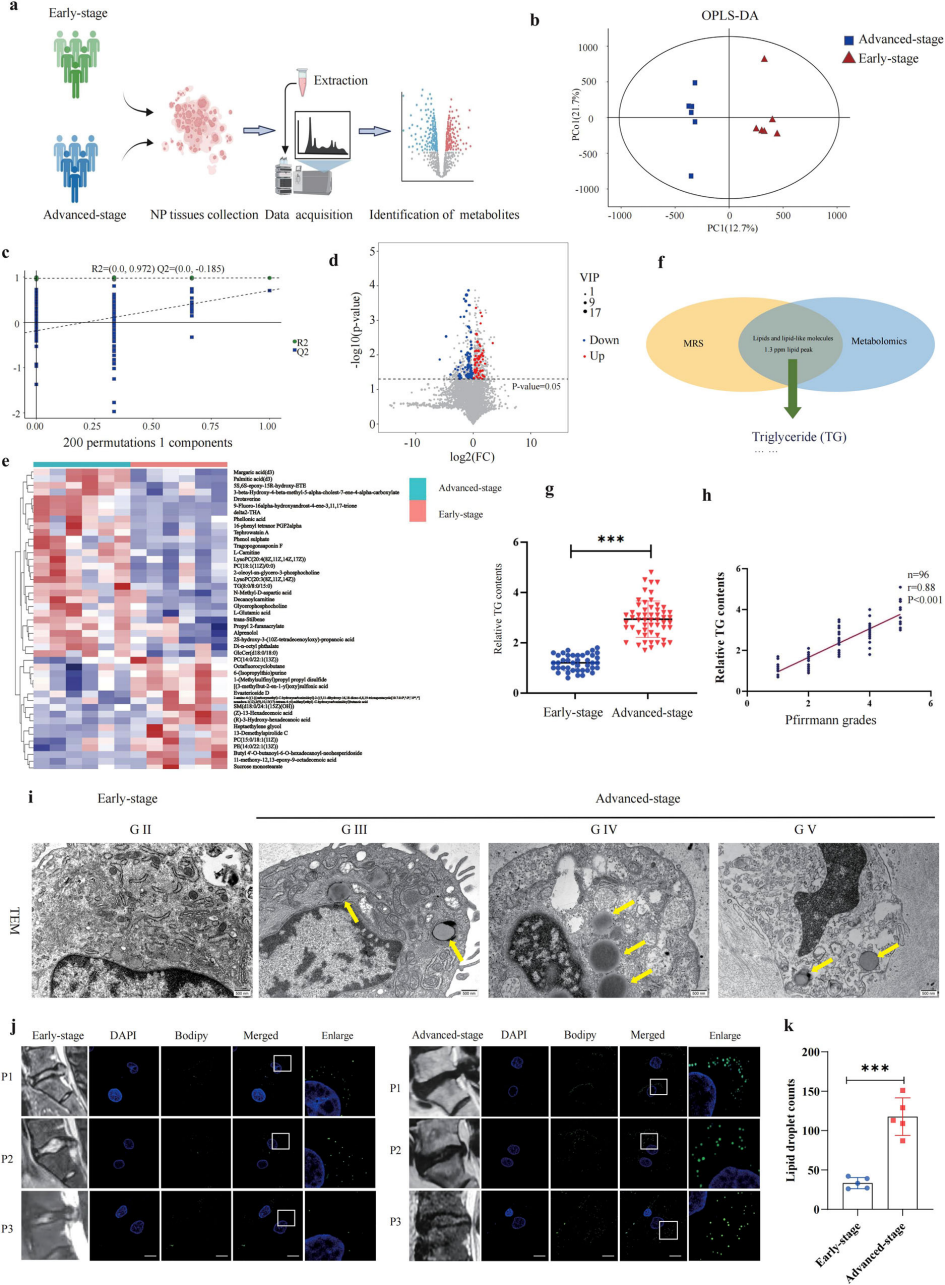
Abnormal intracellular lipid droplet accumulation in IDD. **a** Workflow of metabolomic analysis. **b** Orthogonal partial least squares–discriminant analysis (OPLS-DA) score plots for differentiating the metabolites in each group, the results indicating a clear separation between early- and advanced-stage groups. **c** The corresponding permutation test (200 times) for the OPLS-DA model. **d** Volcano plot showing the differential metabolites with a variable importance projection (VIP) > 1 and *P* < 0.05. **e** Heatmaps of the significantly differential metabolites. **f** Integration of MRS data and metabolomic analysis identified TG levels were significantly altered in human IDD. **g** The relative TG levels of NP cells determined in 41 early-stage and 55 advanced-stage IDD patients. **h** Single-factor linear regression analysis of the relationship between the relative TG contents and Pfirrmann grade. **i** Representative TEM images showing intracellular lipid droplets accumulation in advanced-stage IDD. Scale bar: 2 μm. **j**, **k** BODIPY staining for lipid droplet in patients with early- and advanced-stage IDD. Scale bar: 10 µm. The lipid droplet counts were shown in chart, *n* = 5. ***P < 0.001.

To validate the MRS findings, an LC/MS-based untargeted metabolomics analysis was performed. A chart of the study workflow is shown in Fig. 3a (created with BioRender.com). Samples were collected from 6 patients with early-stage IDD and 6 patients with advanced-stage IDD. Orthogonal partial least squares–discriminant analysis (OPLS-DA) was performed to analyze the data, and the score plots revealed a clear difference in the metabolites between the early- and advanced- stages (Fig. 3b). R2 represents the percentage of the data variance or variation that can be explained by the current model, which indicates the goodness of fit of the model. Theoretically, the closer the R2 values are to 1, the better the model is, while the lower the R2 values are, the worse the fitting accuracy of the model is. In our analysis, the values of R2 = 0.972 obtained by a permutation test indicated a stable analytical platform (Fig. 3c). In total, 45 metabolites were identified with a variable importance projection (VIP) value > 1 and *P* value < 0.05 between the two groups (Fig. 3d, e).

Because MRS analysis revealed abnormal lipid peaks in advanced-stage IDD samples, we focused primarily on lipid-related metabolites and other molecules with levels that we had found to be increased via our metabolomics analysis. Among these molecules, TG was found to be increased (Fig. 3f). NP tissues mainly consist of NP cells and extracellular matrix, but whether TG localizes intracellularly and/or accumulates extracellularly is unknown. In the validation set, NP cells from 41 early-stage and 55 advanced-stage IDD patients were collected to determine the intracellular levels of TG. The total TG level in the advanced-stage NP cells was significantly higher than that in early-stage NP cells (Fig. 3g). In addition, the TG content was positively correlated with disc degeneration grade (*r* = 0.88, *P* < 0.001) (Fig. 3h). A TEM observation and Bodipy staining also revealed intracellular lipid droplet accumulation in advanced-stage IDD samples (Fig. 3i–k). Taken together, the MRS and metabolomics analyses revealed abnormal intracellular lipid droplet accumulation in IDD samples.

### Palmitic acid induces lipid accumulation and NP cell senescence in an ER-stress-dependent manner

**Fig. 4:**
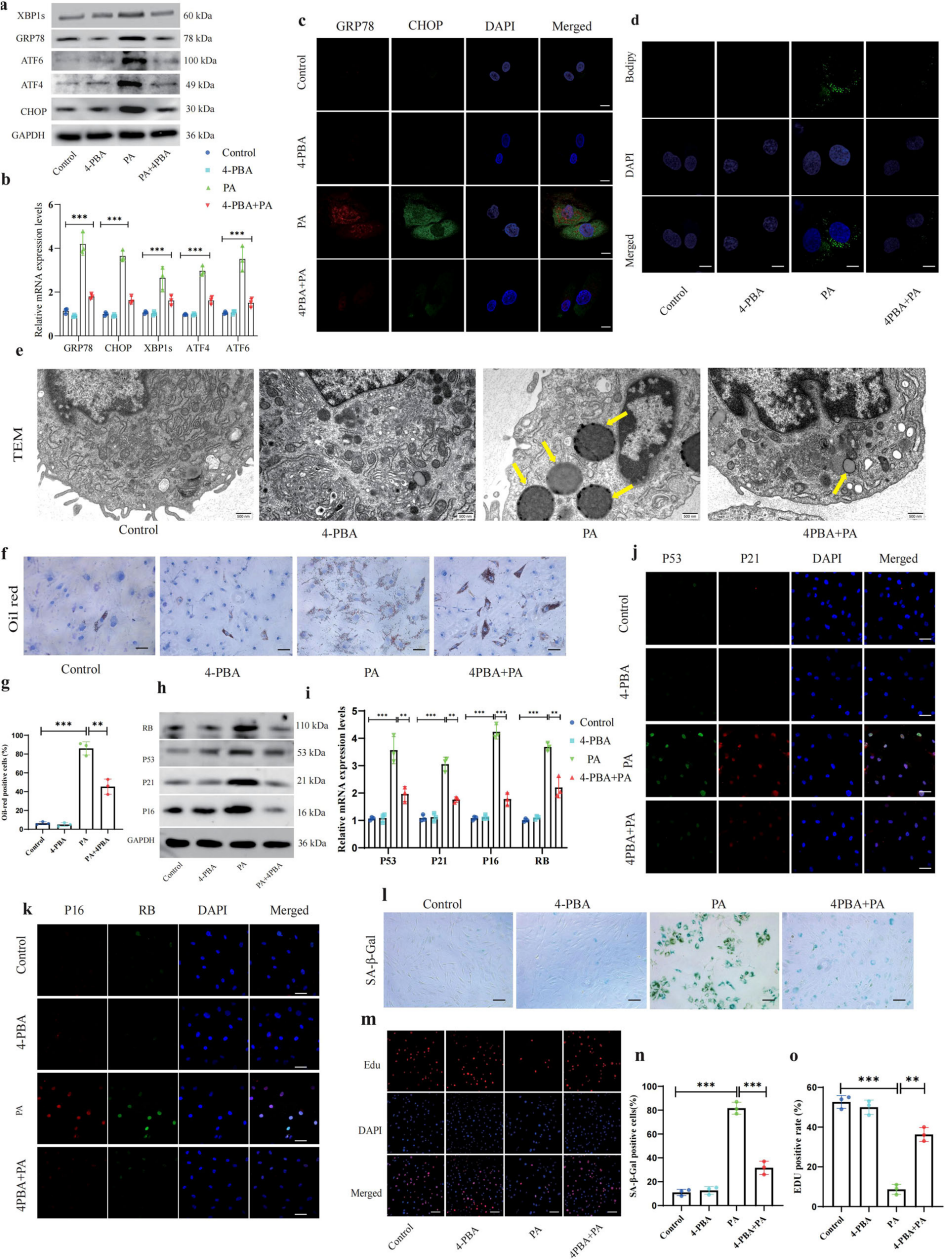
PA induces lipid accumulation and NP cell senescence in an ER-stress-dependent manner. NP cells were incubated with or without the ER-stress-specific inhibitor 4-PBA and 200 μM PA for 24 h. **a**–**c** GRP78 and CHOP protein levels were assessed by WB, PCR, and IF respectively. *n* = 3. **P < 0.01; ***P < 0.001. Scale bar: 10 μm. **d** BODIPY staining for lipid droplets. Scale bar: 10 μm. **e** Ultrastructural view of ER and lipid droplets using TEM. Scale bar: 0.5 μm. **f**, **g** Oil red staining for lipid droplets. Scale bar: 100 μm. The positive oil red staining cells are shown in the statistical chart; *n* = 3. **P < 0.01; ***P < 0.001. **h**–**k** P53, P21, P16, and RB levels were assessed by WB, PCR, and IF respectively. *n* = 3. **P < 0.01; ***P < 0.001. Scale bar: 50 μm. **l** Representative SA-β-gal staining of NP cells. Scale bar: 100 μm. **m** EdU staining detected the proliferation of NP cells. Scale bar: 100 μm. **n**, **o** The positive EdU- and SA-β-gal cells are shown in the statistical chart. *n* = 3. **P < 0.01; ***P < 0.001.

In the LC/MS-based metabolomic analysis, increased palmitic acid (PA) levels in the advanced-stage samples were found. PA is the most common saturated free fatty acid in the body and has been shown to induce ER stress and abnormal lipid metabolism in a number of cell types; however, the relationships among PA, ER stress, lipid droplet accumulation, and NP cell senescence in the IDD context have not been fully elucidated. To investigate the role of ER stress in PA-induced lipid droplet accumulation and cell senescence, NP cells were cultured in the presence of 200 μM PA in the presence or absence of the specific ER-stress inhibitor 4-phenylbutyric acid (4-PBA) at the same for 72 h. WB, PCR, and IF staining showed that the protein levels of GRP78 and CHOP were significantly increased in the PA-treated group compared with those in the control and 4-PBA groups. Additionally, the results indicated that the ER-stress levels were partially suppressed in the presence of 4-PBA (Fig. 4a–c). Lipid droplets were also detected by BODIPY, Oil red, and TEM assays, revealing that PA significantly increased lipid droplet accumulation in NP cells compared with in untreated cells. In contrast, treatment with the ER-stress inhibitor 4-PBA significantly reduced PA-induced lipid droplet accumulation (Fig. 4d–g).

In terms of cell senescence, WB, PCR, and IF analyses showed that the protein levels of P53, P21, P16, and RB were significantly increased in the PA-treated group compared with the control and 4-PBA groups. Additionally, the results indicated that these protein levels were partially suppressed in the presence of 4-PBA, suggesting a role for ER stress in the NP cell senescence pathway (Fig. 4h–k). The results of EdU and SA-β-Gal assays also supported this finding (Fig. 4l–o). These results together demonstrated that ER stress might play an essential role in PA-induced lipid droplet accumulation and NP cell senescence.

### Transcriptomic and proteomic analysis revealed downregulation of SLC43A3 in IDD

**Fig. 5:**
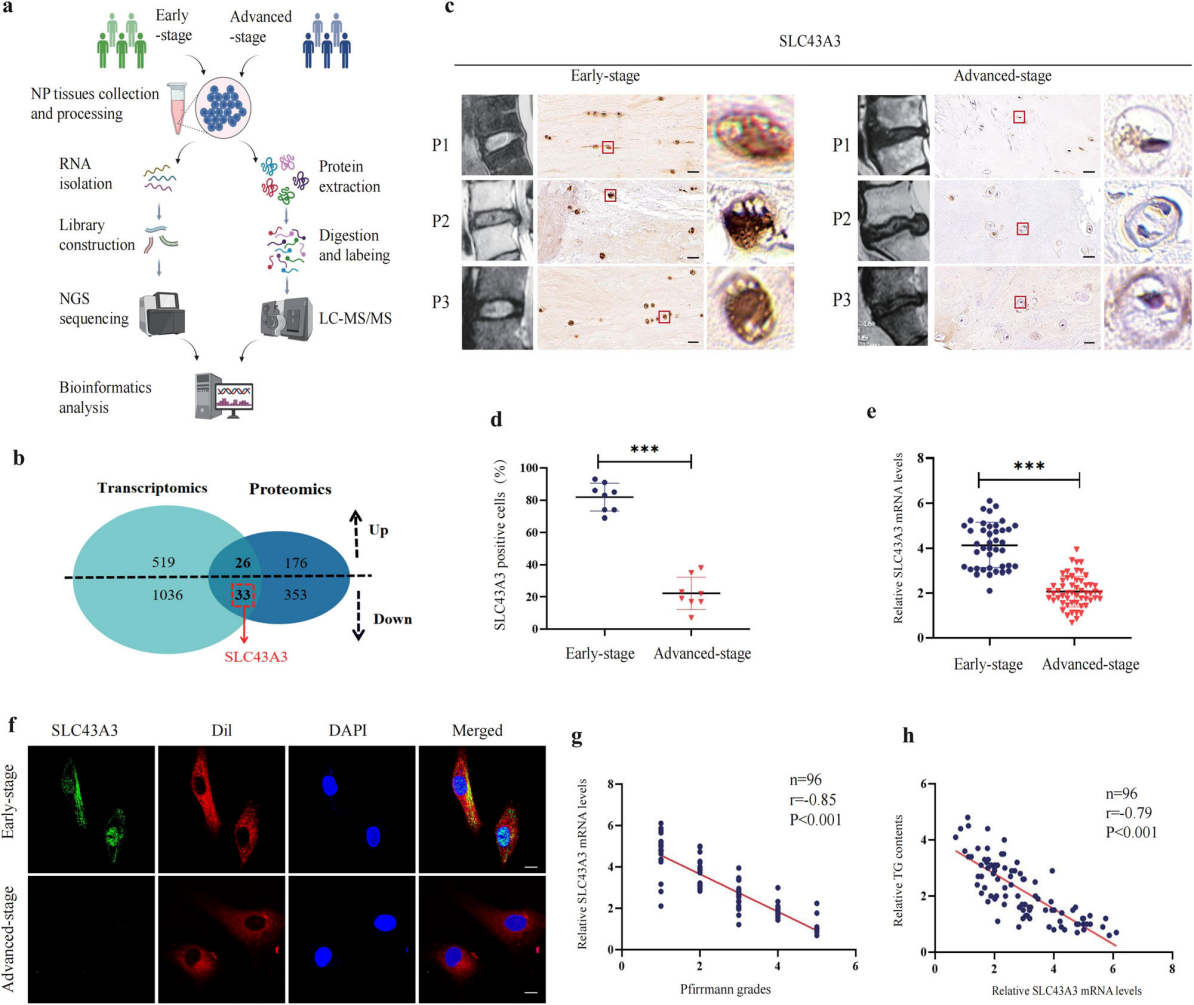
Transcriptomic and proteomic analysis revealed downregulation of SLC43A3 in IDD. **a** Workflow of integrative transcriptomic and proteomic analysis. **b** Integrative analysis identified the downregulation of the SLC43A3 gene and protein level in IDD patients. **c**, **d** IHC staining of SLC43A3 in human NP tissues. Scale bar: 50 μm. The statistical result of positive cell rate was shown in a chart, *n* = 8. ***P < 0.001. **e** The expression level of SLC43A3 mRNA in NP cells was measured in 41 early stage and 55 in advanced stage. ***P < 0.001. **f** IF staining of SLC43A3 in NP cells. Scale bar: 10 μm. **g** The SLC43A3 mRNA expression levels were inversely correlated with the Pfirrmann scores (*r* = −0.85, *P* < 0.001). **h** The TG contents were inversely correlated with the SLC43A3 mRNA expression levels (*r* = −0.79, *P* < 0.001).

Transcriptomic and proteomic data are used to elucidate the mechanisms that drive disease onset and progression and to validate therapeutic targets. In our previous work, the NP samples from 5 patients with early-stage IDD and 5 patients with advanced-stage IDD were collected for transcriptome and proteome profiling. A chart of the study workflow is shown in Fig. 5a (created with BioRender.com). The transcriptomic analysis identified that 1069 genes were downregulated and 545 genes were upregulated between the early and advanced stages. At the protein level, proteomic analysis identified that 386 proteins were decreased and 202 proteins were increased in abundance. Additionally, 59 proteins were found to be significantly altered in the same direction as genes, including 26 upregulated and 33 downregulated (Fig. 5b). The regulation of long-chain free fatty acid levels throughout the plasma membrane relies heavily on a specific transport system. Previous studies have revealed the importance of several proteins, such as FA transport proteins (FATPs), long-chain fatty acyl-CoA synthetases (ACSLs), ABC transporters, and solute carriers (SLCs), in the regulation of free fatty acid uptake and efflux. To find possible IDD-related candidates, we performed an analysis of these 59 overlapped genes and proteins belonging to these abovementioned families.

One of the most interesting genes identified via this transcriptomic and proteomic screen was SLC43A3, which is a positive regulator of free fatty acid efflux and a negative regulator of fatty acid uptake. From the validation dataset, we collected NP tissues from 41 early-stage and 55 advanced-stage IDD patients. The expression of SLC43A3 was significantly downregulated in the advanced-stage IDD samples compared with early-stage IDD samples (Fig. 5c, e). IF staining revealed that the level of SLC43A3, which was distributed mainly distributed throughout the outer cell membrane, was significantly decreased in advanced-stage IDD (Fig. 5f). In addition, the expression of SLC43A3 was negatively correlated with disc degeneration grade (*r* = −0.85, *P* < 0.001) and TG content (*r* = −0.79, *P* < 0.001) (Fig. 5g, h).

### SLC43A3 alleviates ER stress by regulating PA level

**Fig. 6:**
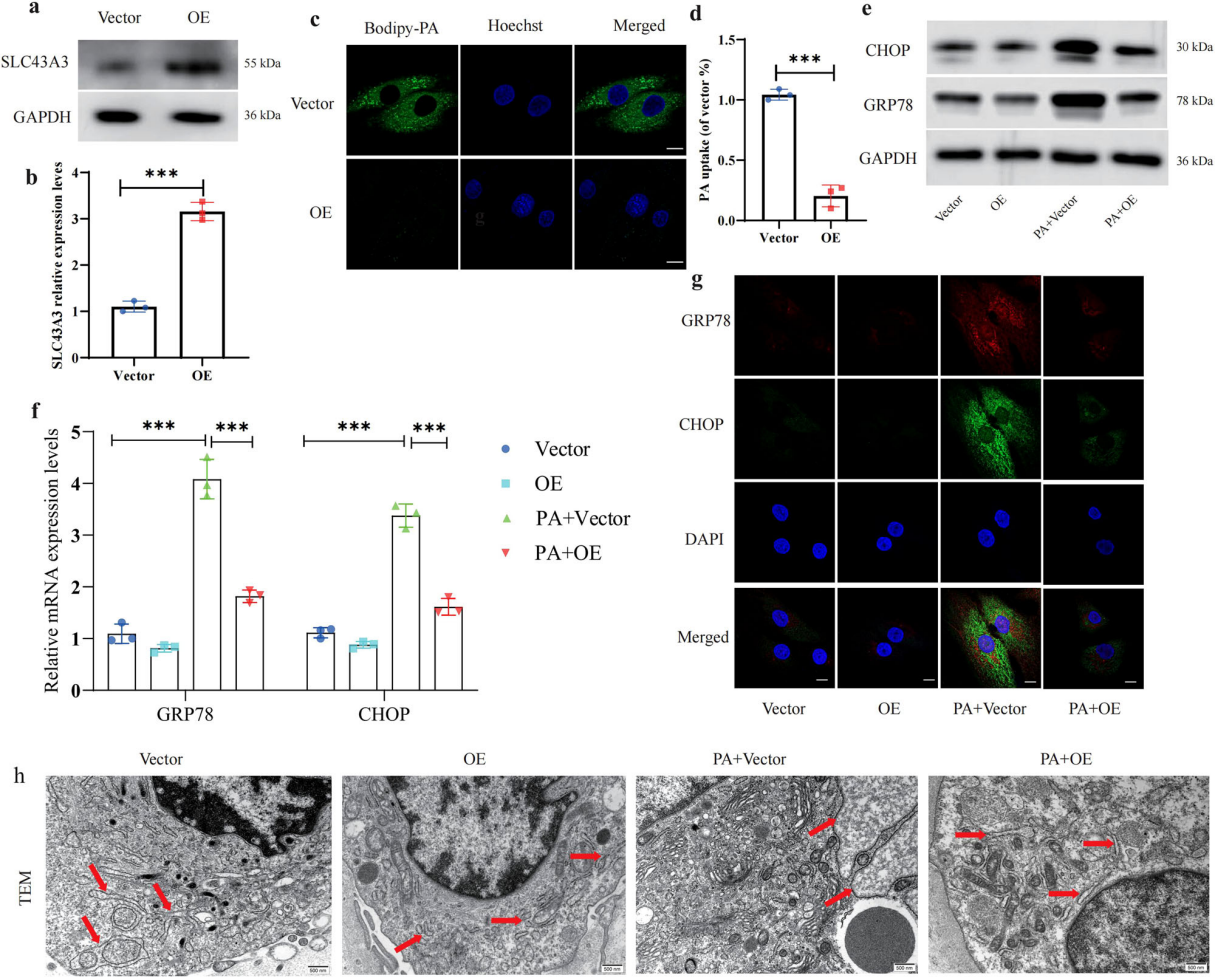
SLC43A3 alleviates ER stress by regulating PA level. **a**, **b** The NP cells in each group were transfected and showed high transfection efficiency. **c**, **d** Uptake of BODIPY-PA in NP cells with or without transfection was determined by measuring the fluorescence intensity. Scale bar: 10 μm. *n* = 3. ***P < 0.001. **e**, **f** The expression levels of ER-stress markers (GRP78 and CHOP) were analyzed by WB and qRT-PCR. *n* = 3. ***P < 0.001. **g** IF staining of GRP78 and CHOP in NP cells after transfection. Scale bar: 10 μm. **h** Ultrastructural view of ER under TEM magnification. Scale bar: 0.5 μm.

Since the functional role of the SLC43A3 gene has not been extensively studied in the pathogenesis of IDD, we examined whether SLC43A3 regulation of PA level truly influences the ER-stress level in IDD tissues. The overexpression efficiency of SLC43A3-carrying plasmids was validated by WB and PCR (Fig. 6a, b). The BODIPY-PA fluorescence was found to be significantly reduced after cell transfection, suggesting that PA uptake was suppressed and that there was a low level of intracellular PA in SLC43a3-transfected cells (Fig. 6c, d). PA treatment induced an obvious increase in GRP87 and CHOP expression levels compared with those in the control cells, but the ER-stress levels were partially decreased after SLC43A3 transfection (Fig. 6f, g). Similar results were found in the alteration patterns of ER stress detected via TEM analysis (Fig. 6h). These results demonstrated that SLC43A3 alleviates ER stress by regulating intracellular PA levels.

### SLC43A3 protects against PA-induced lipid droplet accumulation and cell senescence through the ER-stress pathway

**Fig. 7:**
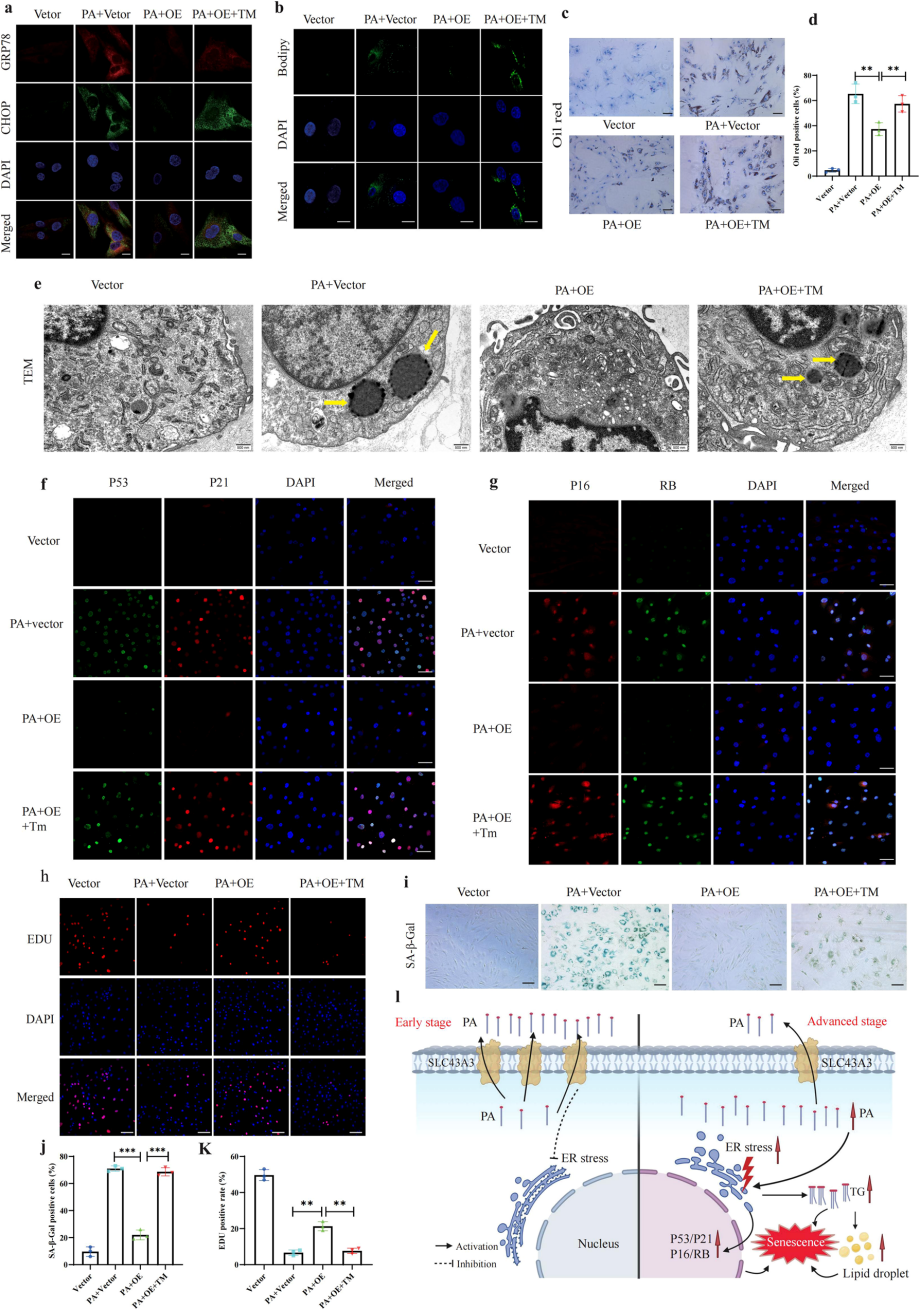
SLC43A3 protects against PA-induced lipid accumulation and cell senescence through the ER-stress pathway. **a** IF staining of GRP78 and Chop in NP cell. Scale bar: 10 µm. **b** BODIPY staining for lipid droplets. Scale bar: 10 μm. **c**, **d** Oil red staining for lipid droplets. Scale bar: 100 μm. The positive oil red staining cells are shown in the statistical chart; *n* = 3. **P < 0.01. **e** Ultrastructural view of lipid droplets using TEM. Scale bar: 0.5 μm. **f**, **g** IF staining of P53, P21, P16, and RB. Scale bar: 50 μm. **h** EdU staining detected the proliferation of NP cells. Scale bar: 100 μm. **i** Representative SA-β-gal staining of NP cells. Scale bar: 100 μm. **j**, **k** The positive EdU- and SA-β-gal cells are shown in the statistical chart. *n* = 3. **P < 0.01; ***P < 0.001. **l** Schematic representation of the mechanism. Abnormal PA accumulation was detected in IDD, resulting in lipid droplet accumulation and cell senescence in an endoplasmic reticulum stress-dependent manner. Overexpression of SLC43A3 significantly alleviated PA-induced endoplasmic reticulum stress, lipid droplet accumulation, and cell senescence by inhibiting PA uptake.

Our results demonstrated that SLC43A3 is an important mediator of PA-induced ER stress. We then analyzed whether SLC43A3 protects NP cells from lipid droplet accumulation and cell senescence induced by ER stress. After SLC43A3 transfection, NP cells were treated with tunicamycin (TM), which disrupts protein glycosylation to induce ER stress. SLC43A3 significantly attenuated lipid droplet accumulation after PA stimulation, but treatment with TM blocked the effect of SLC43A3 (Fig. 7a–c). A TEM observation revealed similar results (Fig. 7d). Additionally, SLC43A3 decreased the levels of P53, P21, P16, and RB, but the levels of these senescent markers were increased to a certain degree in the presence of TM (Fig. 7e, f). Cell senescence was also detected by EdU and SA-β-Gal assays, revealing that SLC43A3 significantly decreased the rate of cells senescence, but treatment with TM hindered the protective effect of Slc43A3 treatment (Fig. 7g–j). These results together indicated that SLC43A3 protects against PA-induced lipid droplet accumulation and cell senescence induced through the ER-stress pathway (Fig. 7k) (created with BioRender.com).

### Slc43A3 inhibited disc degeneration in the IDD model by ameliorating ER stress, lipid accumulation, and cell senescence

**Fig. 8:**
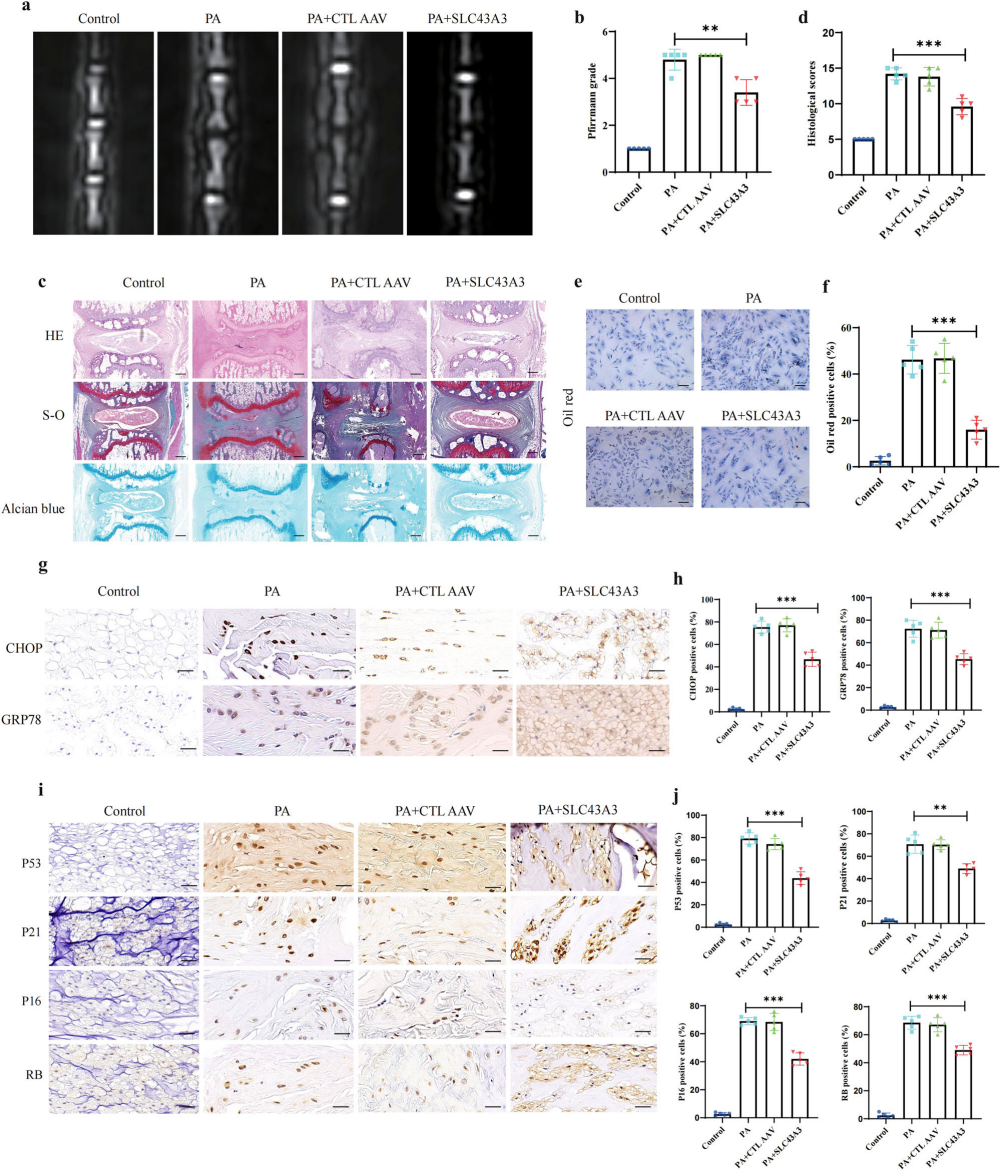
SLC43A3 inhibited disc degeneration in the IDD model. **a**, **b** Representative MR T2-weighted images of rat tail at eight weeks post needle puncture and degenerated degrees were evaluated according to the Pfirrmann system. *n* = 5. ***P < 0.001. **c**, **d** HE, Alcian blue, and S-O staining of disks eight weeks postoperatively; Scale bar: 1 mm. The grading scores of histological analysis are shown in the statistical chart. *n* = 5. ***P < 0.001. **e**, **f** Oil red staining for rat NP cell. Scale bar: 100 μm. The positive oil red staining cells are shown in the statistical chart; *n* = 3. **P < 0.01; ***P < 0.001. **g**, **h** IHC staining for GRP78 and CHOP at eight weeks after needle puncture. Scale bar: 50 μm. The rates of positive cells were calculated; *n* = 5. ***P < 0.001. **i**, **j** IHC staining for P53, P21, P16, and RB at eight weeks after needle puncture. Scale bar: 50 μm. The rates of positive cells were calculated; *n* = 5. **P < 0.01; ***P < 0.001.

To assess the pathogenic role of PA in IDD, we constructed an animal model with Sprague-Dawley rats. T2-weighted MR images demonstrated lower Pfirrmann scores in the PA group than in the control group, indicating severe disc degeneration, but the scores were significantly increased after the model rats were treated with SLC43A3 (Fig. 8a, b). In addition, as determined by HE, S-O and Alcian blue staining, the PA group presented with obviously narrowed disc space, annular layer disorganization and inward bulging of the inner annulus, whereas SLC43A3-treated rats showed significantly ameliorated the destruction of disc cellularity and morphology (Fig. 8c, d). Lipid droplet accumulation was evaluated using an oil red stain, which revealed that the NP cells in the PA+IDD models presented a high degree of lipid accumulation, with these results partially reversed following treatment with SLC43A3 (Fig. 8e, f). Moreover, IHC staining indicated that treatment with SLC43a3 alleviated the expression of GRP78 and CHOP compared to the expression levels in the PA group, thereby confirming our in vitro results (Fig. 8g, h). An IHC analysis of P53, P21, P16, and RB levels also suggested that SLC43A3 attenuated NP cell senescence during IDD progression (Fig. 8i, j). Together, these results demonstrated the impact of PA on IDD via ER stress, lipid droplet accumulation, and the cell senescence pathway and suggested that inhibiting the accumulation of PA may be a promising strategy for IDD treatment.

## Discussion

A variety of intracellular stress conditions have been recognized as predisposing factors for ER stress, which initiates a set of intracellular pathogenic responses that interfere with the normal function of NP cells and ultimately result in IDD^13,16,23,24^. Among these stress conditions, toxic metabolite overload has gradually attracted increasing attention^23^. Previous studies have documented the role of various metabolites, such as reactive oxygen species, free fatty acids, and advanced glycosylation end products, in IDD pathogenesis with action mediated via the ER-stress pathway^13,24^. The intervertebral disc is an avascular organ that is relatively closed in vivo, and all nutrient-supplied and metabolic wastes removed are slowly diffused through the cartilage endplate; therefore, the microenvironment homeostasis of NP cells is highly susceptible to metabolic profile alterations^25^. However, relatively little information regarding overall metabolic profile alteration due to NP cell senescence is available, which has limited the ability to gain further insights into the predisposing factors of ER stress in IDD. In addition, the ER is an important intracellular organelle critical to the biosynthesis of steroids, lipids, and carbohydrates; therefore, perturbation of ER homeostasis changes the metabolic profile^12^. Therefore, an analysis of global metabolic profile alterations during the course of IDD may provide data-rich information that reflects the stress conditions influencing ER homeostasis, which can be used to clarify the complex regulatory relationships among stress conditions, ER stress, and NP cell senescence.

MRS is a noninvasive technique based on the magnetic properties of atomic nuclei and can be used for the quantitative analysis of the biochemical composition in the living human body. In clinical practice, MRS has been extensively used to characterize in vivo metabolic features within NP and other types of tissues^26–28^. Therefore, to monitor real-time metabolic profile alterations in IDD patients, the MRS technique was employed in the current study. The MRS spectra obtained from nondegenerative intervertebral disks of both healthy volunteers and IDD patients were similar to those previously observed for healthy NP tissues. In contrast, spectra acquired from the degenerated NP tissues of IDD patients featured a higher lipid peak at approximately 1.3 ppm, indicating abnormal lipid accumulation^26^. Our MRS findings with the IDD samples were not completely consistent with previous observations from surgically harvested samples and in vivo validation studies^28^. Using ex vivo MRS, for example, Keshari et al. ^29^ showed that certain intradiscal metabolites, specifically lactate and proteoglycan, were quantifiable metabolic biomarkers for discogenic pain and intervertebral disc degeneration. A subsequent in vivo intervertebral disc MRS analysis revealed different lactate and proteoglycan contents when the intervertebral disks were clinically categorized into a painful group and a nonpainful group^26^. The differences between previous studies and our work may be partially explained by different samples of enrolled patients. In the study conducted by Gornet et al., patients who were diagnosed with discogenic pain were included in the MRS analysis, while those with spondylolisthesis, scoliosis, and disc herniation were excluded. In the present study, the study advanced-stage study cohort consisting of 3 individuals with disc herniation and 2 individuals with spondylolisthesis.

The lipid family is composed of a wide range of molecular species, including TGs, sphingolipids, phosphoglycerides, and sterols^30^. To increase the interpretability of the MRS analysis, we performed an untargeted LC/MS-based metabolomic analysis with surgically harvested NP tissues. We found that the abundance of 14 metabolites was increased and that of 41 metabolites was decreased in advanced-stage IDD compared with early-stage IDD. Because the MRS analysis suggested abnormal lipid accumulation in advanced-stage IDD, a specific focus was placed on an analysis of lipids and lipid-related molecules identified in our metabolomics profile. Notably, the TG contents were significantly higher in the advanced-stage IDD samples. This finding was subsequently verified with data from another cohort of IDD patients that showed intracellular lipid droplet accumulation. In atherosclerosis samples, lipid droplet-enriched cells showed the expression of senescent markers, and their expression seemed to indicate a deleterious effect throughout all stages of disease^31^. Similarly, lipid droplets in microglia have been previously identified in the context of brain aging and disease^32^. However, lipid droplet accumulation has not been functionally studied in NP cells, and little is known about the formation and role of lipid droplets in IDD. By integrating MRS and untargeted LC/MS-based metabolomics with validation of the findings with large-cohort data, our results suggest that the spectroscopically identified lipid markers may be useful metabolic markers for the investigation of IDD.

Another key finding from the metabolomics profile revealed that PA level was largely unregulated in the advanced-stage IDD sample. PA is one of the most common free fatty acid components found in the human body and has been shown to induce ER stress and cell dysfunction^7,33^. Previous studies have revealed that pharmacologic ER-stress conditions increase de novo lipogenesis and lipid droplet accumulation in hepatic steatosis by upregulating genes encoding key lipogenic transactivators, enzymes, and pathways^34^. For example, Jo et al. ^35^ demonstrated that activation of the PERK-eIF2α-ATF4 pathway under ER-stress conditions was required for inducing the upregulation of very low-density lipoprotein receptor, which is critical for intracellular lipid droplet accumulation and hepatic steatosis, in hepatocytes. Similarly, another targeted metabolomics study revealed that 2,3,7,8-tetrachlorodibenzofuran exposure induced ER stress, which ultimately led to hepatic steatosis and altered phospholipid and choline metabolism^20^. In the present study, PA treatment upregulated the levels of the ER-stress markers GRP78 and CHOP and lipid droplet accumulation; moreover, PA-induced lipid droplet accumulation was markedly reduced following treatment with the ER-stress-specific inhibitor 4-PBA. According to the integrated analysis of the MRS data and metabolomics profiles, our preliminary findings verified a potential role for PA in ER stress and lipid droplet accumulation, which has been identified in IDD phenotypes.

Lipid droplet accumulation is closely related to tissue dysfunction in a variety of human diseases, including nonalcoholic fatty liver disease, insulin-resistant skeletal muscle, atherosclerosis, and diabetic cardiomyopathy^19,31,36,37^. Chronic accumulation of lipid droplets can compromise cellular functions via multiple mechanisms, such as activation of the inflammatory pathway through an increase in proinflammatory cytokine and Toll-like receptor levels, ER-stress responses, and oxidative stress responses through the mitochondrial pathway and the induction of cellular senescence and death signaling^32,38^. In the current study, as evidenced by the findings of the MRS analysis and metabolomics profiling with validation of the finding with large-cohort data, intracellular lipid droplet accumulation can be considered a biomarker of IDD progression. The clinical significance of the present work provides a basis for the potential development of a noninvasive in vivo MRS approach to quantify biomarkers of IDD.

The levels of intracellular free fatty acids are dynamically regulated in response to multiple physiological and pathological factors. In summary, short-chain and medium-chain free fatty acids show high membrane permeability and thus can easily diffuse across the plasma membranes of cells without specific transporters needed for their uptake or efflux; however, long-chain free fatty acids, such as PA detected in our metabolomics profile, show limited permeability across the plasma membrane^39^. Regulation of long-chain free fatty acids across the plasma membrane relies heavily on a high-affinity and low-capacity transporter system^40^. Membrane proteins appear to be involved in this long-chain free fatty acid transport via a rapid, saturable, substrate-specific mechanism. Previous studies have demonstrated the important role of several protein families, such as solute carriers (SLCs), FA transport proteins (FATPs), ABC transporters, and long-chain fatty acyl-CoA synthetases (ACSLs), in the protein-facilitated process of free fatty acid uptake and efflux^41–43^. However, the causes of abnormal intracellular PA aggregation in IDD are incompletely understood. Therefore, a deeper understanding of the molecular mechanisms involved in the abnormal intracellular PA aggregation will likely be of great therapeutic value for IDD.

The integration of metabolomic profile data with other omics data, such as transcriptomic and proteomic profile data, is becoming an increasingly common approach to fully exploit outcomes and increase result interpretability^18,21^. Theoretically, both metabolomics and transcriptomic profiles show some limitations. Metabolomics analysis, for example, provides only a phenotypic overview of a disease, while transcriptome analysis is limited by the research on specific genes, meaning the results may not show subsequent changes in translation or posttranslational modifications. Through integration with transcriptomic or proteomic profile data, the results from a metabolomic profile are more comprehensive and persuasive^17^. In the study of Ma et al. ^19^, the combination of metabolomic and transcriptomic profiles provided reliable information about liver pathology, including key pathways, potential metabolic markers, and genes involved in liver ER stress. Similarly, with an integrative approach, Yuan et al. ^20^ identified signature genes, including the aryl hydrocarbon receptor, that are involved in phospholipid and choline metabolism in hepatic steatosis and thus suggested new therapeutic strategies for fatty liver disease.

Using an integrative transcriptome and proteome profiling approach, we detected many differentially expressed genes involved in lipid uptake and efflux. With a focus on plasma membrane-localized transporters, we found that the gene encoding the SLC43A3 solute carrier to be particularly interesting. Moreover, validation with data obtained from a large-cohort sample showed that SLC43A3 expression levels were negatively correlated with lipid content and disc degeneration grade. SLC43A3 has been functionally identified as a positive regulator of free fatty acid efflux and a negative regulator of free fatty acid uptake. In adipocytes, knockdown of SLC43A3 significantly reduced both basal and forskolin-stimulated FFA efflux while increasing free fatty acid uptake and lipid droplet accumulation^39^. Our results were in line with the findings of the abovementioned study, demonstrating that the overexpression of SLC43A3 significantly decreased intracellular lipid droplet accumulation. Since the functional role of the SLC43A3 gene has not been studied in the pathogenesis of IDD, our study was also aimed at determining whether SLC43A3 prevents PA-induced ER stress in NP cells, and the results revealed that overexpression of SLC43A3 significantly decreased the expression of the ER-stress markers GRP78 and CHOP. Therefore, these results indicated that the downregulation of SLC43A3 plays a protective role in PA-induced ER stress in NP cells.

In the present work, we applied MRS- and LC/MS-based metabolomics analysis to characterize metabolic features during the course of IDD for the first time. This metabolomic analysis provides key findings on the causes of ER stress and further explains the pathomechanisms of intracellular lipid droplet accumulation and NP cell senescence. A feasible reason for PA accumulation might be the downregulation of SLC43A3, which we detected in this work by transcriptome profiling. SLC43A3 is required for the influx of free fatty acids, increasing their intracellular level, thus being directly involved in the activation of ER stress. Our other investigations demonstrated that the upregulation of SLC43A3 expression promoted a marked decrease in free fatty acids and alleviated intracellular lipid droplet accumulation and NP cell senescence in vitro. Additionally, our data revealed that the lipid droplet content and ER-stress level in NP cells were lower in PA+SLC43A3 rats than in IDD rats; moreover, the degenerative changes in IDD rats were partially ameliorated by the administration of SLC43A3. Collectively, our findings from the integration of multiomics data provide a systems-level perspective of IDD that had been previously unrecognized. Of translational significance, the alterations in lipid metabolism reported herein may facilitate the development of novel biomarkers and therapeutic targets for IDD.

Although our findings are novel, several limitations are to be noted. First, the number of samples used for metabonomics analysis was relatively small, and therefore, the metabonomic profile obtained may only partially reflect the diverse metabolic alterations and pathogenic pathways in IDD-related cells. However, most importantly, by combining omics data with large-cohort validation and functional assessment data, our study provides mechanistic insights into the ER stress, lipid droplet accumulation, and NP cell senescence that we observed in IDD. Second, the molecular alterations in response to these metabolic alterations were influenced by a combination of multiple mechanisms; therefore, the incorporation of proteomic profiling data may further deepen our understanding of abnormal lipid metabolism in IDD.

IDD is a prominent cause of persistent low back pain, posing significant challenges to public health due to its substantial societal, economic, and familial burdens. Given the limited efficacy of current therapeutic approaches, the imperative to develop targeted interventions for this condition is of paramount importance. ER stress is widely recognized as a prominent characteristic of pathological conditions associated with IDD. The avascular and confined nature of intervertebral disks renders them particularly susceptible to the accumulation of harmful metabolites, thereby increasing their vulnerability to ER stress. Additionally, the regulation of intracellular metabolite levels is influenced by various factors, including genes and proteins. The present study adopts the following approach^1^: The integration of metabolomics and MRS analysis is employed to investigate metabolite alterations associated with ER stress. We found abnormal PA accumulation in IDD nucleus pulposus cells, and PA exposure resulted in lipid droplet accumulation and cell senescence in an endoplasmic reticulum stress-dependent manner^2^. Transcriptomic and proteomic integration is utilized to identify regulated networks. We detected that SLC43A3 was expressed at low levels and negatively correlated with intracellular lipid content in IDD nucleus pulposus cells. Overexpression of SLC43A3 significantly alleviated PA-induced endoplasmic reticulum stress, lipid droplet accumulation, and cell senescence by inhibiting PA uptake. Importantly, our investigation provides novel insights into the mechanistic role of the SLC43A3-PA-ER stress axis in the senescence of NP cells through the vertical integration of multiomics data, along with functional assessments.

In summary, our MRS- and untargeted LC/MS-based metabolomics analysis enabled us to identify the enrichment of lipid molecules in IDD, which was important for underscoring the pivotal role played by PA in the pathogenesis of disease and acquisition of disease-related phenotypes. Our results also enabled us to identify spectroscopic markers of lipids that may serve as metabolic markers of IDD, suggesting a basis for the development of a noninvasive in vivo MRS approach to the investigation of IDD progression and treatment. In the discovery process of these metabolic alterations, SLC43A3 was identified as a functional positive regulator of free fatty acid efflux and a negative regulator of free fatty acid uptake, laying a foundation for therapeutic target validation. These findings might enhance the understanding of the pathogenesis of IDD and lead to a new treatment strategy against IDD in the future.

## Methods

### Patient samples

This study was approved by the ethics committee of our institution, and written informed consent was obtained from each patient. NP samples were obtained from patients who received discectomy due to neurological deficits, cauda equina syndrome and/or low back pain. The degree of human NP tissue sample degeneration was graded using Pfirrmann grades applied on the basis of MR T2-weighted images: Grades I–II were collectively defined as early-stage degeneration, and Grades III–V were collectively defined as advanced-stage degeneration. NP tissues were cut into pieces and enzymatically digested in 0.25% Type II collagenase for 4 h at 37 °C. After isolation, the NP cells were resuspended in DMEM (Gibco) supplemented with 10% FBS, 100 U/ml penicillin, and 100 mg/ml streptomycin and maintained at 37 °C in a humidified atmosphere with 95% atmospheric air and 5% CO^2^. The medium was changed every 3 days. The NP cells from the second passage were used for the subsequent experiments.

### Magnetic resonance spectroscopy (MRS) analysis

MRS allows noninvasive assessment of metabolic alterations within a tissue of interest in vivo and has been proven to be an extremely powerful and accurate analytical technique for metabolomics research^26–28^. Therefore, in the current study, an MRS analysis was performed to characterize the in vivo metabolic features of the IDD NP in patients. Written informed consent was obtained from each patient for MRS and Ethics Committee approval from the First Affiliated Hospital of USTC was obtained. The examinations were performed using a 3.0 T magnetic resonance scanner (GE, USA) with the subject in a supine position. Five healthy volunteers and 5 IDD patients were examined. The advanced-stage group consisted of 3 patients with disc herniation and 2 patients with spondylolisthesis. For regions of interest in the sagittal, coronal, and axial planes, the scanner operator prescribed a single voxel (for SVS) that encompassed a disc nucleus and excluded the vertebral body. Lumbar spinal MRI was performed according to the routine procedure, and MRS data were collected as part of a secondary MRI session after routine clinical MRI was performed^26^. After MRS voxel positioning, scanning was performed via single-voxel proton MRS with 1000 ms TR and 37 ms TE. The MRS data were not utilized for surgery.

### Metabolomics-based sequencing

An LC/MS-based untargeted metabolomic analysis was performed by Ouyi Biotech Ltd. according to the standard protocol (Shanghai, China). After protein extraction and quality control, an ACQUITY UHPLC system (Waters Corporation, Milford, USA) coupled with an AB TripleTOF 6600 Plus system (AB SCIEX, Framingham, MA) was used to obtain the metabolite profile in both ESI positive and ESI negative ion modes. The binary gradient elution system consisted of (A) water (containing 0.1% formic acid, v/v) and (B) acetonitrile (containing 0.1% formic acid, v/v), and separation was achieved by using the following program: 5% B; 2 min, 20% B; 4 min, 60% B; 11 min, 100% B; 13 min, 100% B; 13.5 min, 5% B and 14.5 min, 5% B. The LC flow rate was 0.4 mL/min, and the column temperature was 45 °C. All of the samples were maintained at 4 °C during the analysis. The MS flow rate was 0.35 mL/min, and the injection volume was 2 µL. Data acquisition was performed in full scan mode from 70 to 1000 m/z in information-dependent acquisition mode. The parameters for mass spectrometry were as follows: ion source temperature, 550 °C (+) and 550 °C (−); ion spray voltage, 5500 V (+) and 4500 V (−); curtain gas, 35 PSI; declustering potential, 100 V (+) and − 100 V (−); collision energy, 10 eV (+) and − 10 eV (−); and interface heater temperature, 550 °C (+) and 600 °C (−). For an IDA analysis, the scan range was set to be m/z 25–1000, and the collision energy was 30 eV.

The LC/MS raw data were extracted and processed with Progenesis QI software (Waters Corporation, Milford, MA, USA). The parameters were set as follows: a precursor tolerance of 5 ppm, a fragment tolerance of 10 ppm, and a production threshold of 5%. The resulting matrix was used to construct three-dimensional datasets that included retention time, mass-to-charge ratio (m/z), and normalized ion intensities. The matrix was reduced by removing peaks for which values were missing (ion intensity = 0) in more than 50% of the samples. Metabolites were identified by Progenesis QI (Waters Corporation, Milford, USA) data processing software based on public databases such as http://www.hmdb.ca/; http://www.lipidmaps.org/and in-house databases (Ouyi Corporation, Shanghai, China). Both the positive and negative selection data were combined and imported into R software. Orthogonal partial least squares–discriminant analysis (OPLS-DA) was carried out to visualize differences between groups. The criteria for identifying significant differentially abundant metabolites were variable importance in the projection (VIP) value > 1 and *P* value < 0.5 in the OPLS-DA model.

### Transcriptomic sequencing

Transcriptomic sequencing was performed by Ouyi Biotech (Shanghai, China). Total RNA was isolated from NP cells from early- and advanced-stage IDD samples using TRIzol reagent. After quality control, libraries were constructed using a TruSeq Stranded mRNA LT Sample Prep Kit (Illumina, USA). Then, these libraries were sequenced on an Illumina HiSeq X Ten platform, and 150-bp paired-end reads were generated. The raw reads in fastq format were first processed by Trimmomatic, and poly-N and low-quality reads were removed to obtain clean reads. Then, the clean reads were aligned to the human reference genome using HISAT2. The fragments per kb per million reads (FPKM) value of each gene was measured by using cufflinks, and the counts per gene were obtained using htseqcount. Differential expression analysis of the two groups was performed using the DESeq (2012) R package. Significantly differently expressed genes reached a difference threshold with *P* value < 0.05 and a fold change >2 or a fold change <0.5. A hierarchical clustering analysis was performed to identify distinguishable expression patterns between the early- and advanced-stage degeneration groups.

### Proteomics sequencing

Proteomic sequencing was performed by Ouyi Biotech (Shanghai, China). The NP cells were lysed in lysis buffer supplemented with 1 mM PMSF. Then, the samples were homogenized on ice and further lysed with sonication at 1 s/1 s intervals for 3 min at 80 W power. Cell debris was removed by centrifugation at 12,000 × *g* for 10 min at 4 °C. The protein concentration was determined by a BCA protein assay kit. Protein digestion and TMT labeling were performed according to the manufacturer’s protocol (Thermo Scientific, USA). In brief, 100 µg of protein extract was reduced, alkylated, and digested with trypsin at 37 °C for 12 h. After digestion, the peptide was desalted and vacuum-dried. Next, the peptide was reconstituted in 0.5 M TEAB and processed according to the protocol for the TMTpro 16 kit. Then, the TMT-labeled peptides were fractionated on an 1100 HPLC system (Agilent) using an Agilent Zorbax Extend RP column (5 μm, 150 mm * 2.1 mm) with 215 nm and 280 nm UV monitions. Peptide separation was performed at 300 µl/min using a nonlinear binary gradient starting with phases A (2% acetonitrile in HPLC water) and B (98% acetonitrile in HPLC water). The samples were collected for 8–60 min, and the eluent was collected in a centrifuge tube 1–15 every minute. The separated peptides were dried for mass spectrometry.

LC‒MS/MS analysis was performed by a Q-Exactive system (Thermo Scientific, USA). The samples were loaded onto an Acclaim PepMap100 100 μm × 2 cm (RP-C18, Thermo Fisher) and separated by an Acclaim PepMap RSLC system (75 μm × 50 cm, RP-C18, Thermo Fisher). The flow rate was 300 nL/min, and the linear gradient was 75 min (0 ~ 63 min, 5 ~ 45% B; 63 ~ 65 min, 45-90% B; 65 ~ 75 min, 90% B; mobile phase A = 0.1% formic acid in water and B = 0.1% FA in acetonitrile). Full MS scans (m/z range 350–1500) were acquired with a resolution of 60,000, and the AGC target was set to 3E6. The 20 most intense peaks in the MS spectra were fragmented with higher-energy collisional dissociation (HCD) with a collision energy of 32. MS/MS spectra were obtained at a 45,000 resolution with an AGC target of 2e5 and a max injection time of 80 ms. The dynamic exclusion was set to 30.0 s and run under positive mode.

Proteome Discoverer (v.2.4, Thermo, USA) was used to search all of the raw data thoroughly against the sample protein database. Database searches were performed with trypsin digestion specificity. Cysteine alkylation and TMT addition were considered fixed modifications in the database search. For protein quantization, it was required that a protein contains at least 2 unique peptides, with a false discovery rate (FDR) < 0.01. Identified proteins with a *P* value  ≤  0.05 and fold change ≥1.2 or ≤0.83 were considered to be upregulated and downregulated, respectively.

### Western blot analysis (WB)

Total proteins from human NP tissues or NP cells were lysed using RIPA lysis buffer (Beyotime, China) according to standard methods. Then, proteins were electrophoresed in 10% or 12% SDS‒PAGE gels and transferred to PVDF membranes. Membranes were blocked with 5% nonfat dried milk for 2 h and incubated with anti-GRP78 (Zenbio, China; 1:1000 dilution), anti-CHOP (Proteintech, China, 1:1000 dilution), anti-P53 (Servicebio, China; 1:1000 dilution), anti-P21 (Zenbio, China; 1:1000 dilution), anti-P16 (Cohesionbio, UK; 1:1000 dilution), anti-RB (CST, USA; 1:1000 dilution) and anti-SLC43A3 (Santa, USA, 1:1000 dilution) antibodies at 4 °C overnight. After washing with TBST, the membranes were incubated with horseradish peroxidase (HRP)-conjugated secondary antibodies and visualized using an ECL reagent (Beyotime Biotech, China). GAPDH expression was used for normalization.

### Quantitative real-time reverse transcription-PCR (qRT-PCR)

Total RNA was extracted with Trizol reagent (Invitrogen, USA) from cultured NP cells, reverse‐transcribed, and amplified by qRT-PCR according to the instructions. The primers used for qRT-PCR were as follows: P53(forward, CAGCACATGACGGAGGTTGT; reverse, TCATCCAAATACTCCACACGC), P21(forward, TGTCCGTCAGAACCCATGC; reverse, AAAGTCGAAGTTCCATCGCTC), P16(forward, GATCCAGGTGGGTAGAAGGTC; reverse, CCCCTGCAAACTTCGTCCT), and RB(forward, CTCTCGTCAGGCTTGAGTTTG; reverse, GACATCTCATCTAGGTCAACTGC), SLC43A3(forward, CCGCCACACTCATCATAGCC; reverse, GGCCAAATAGGTTCCCAATCTG), GRP78(forward, CATCACGCCGTCCTATGTCG; reverse, CGTCAAAGACCGTGTTCTCG), CHOP(forward, GGAAACAGAGTGGTCATTCCC; reverse, CTGCTTGAGCCGTTCATTCTC). Expression levels of target genes were normalized to GAPDH expression. Fold changes in gene expression were calculated by the comparative cycle threshold (CT) method using the formula 2-(ΔΔct).

### Triglyceride (TG) assay

The intracellular TG level was assayed using a triglyceride assay kit (Solarbio, China) according to the manufacturer’s instructions. A total of 1 × 10^5^ cells were harvested for measuring the total TG content. The TG content was normalized to that of the respective control or untreated cells.

### Cell transfection

An SLC43A3-overexpressing plasmid and control vectors were obtained from Shanghai Genomeditech Company (Shanghai, China). To overexpress SLC43A3, full-length human SLC43A3 cDNA was cloned into a lenti-CMV-MCS-PGK-Puro vector. Briefly, NP cells were seeded in 35-mm dishes and cultured overnight, and then, plasmids were transfected into the NP cells using Lipofectamine 2000 transfection reagent (Invitrogen) and Opti‐MEM (Gibco). Before further experiments were performed, the efficacy of transfection was measured by WB and qRT-PCR.

### Histology and immunostaining assays

Human NP tissue samples were fixed in 4% paraformaldehyde, embedded in paraffin, sectioned into 5-μm sections, and stained with hematoxylin and eosin (HE), Masson, and Alcian blue according to the typical respective protocols. Animals were euthanized, and the disks were harvested, fixed with paraformaldehyde for 24 h, decalcified slowly with 10% EDTA for 4 weeks, dehydrated, embedded in paraffin, and sectioned into 5-μm slices. The slices of each disc were stained with HE, safranin O, fast green (S-O), and Alcian blue. The degree of disc degeneration was assessed on a histological grading scale based on the evaluation of the annulus fibrosus, endplate, and NP tissues, with 5 points representing a relatively normal disc and 15 points representing a severely degenerated disc^44^.

Immunohistochemical staining (IHC) of 5-µm thick slices from human or animal samples was performed as described previously. Briefly, the slices were deparaffinized and rehydrated and then microwaved in sodium citrate for 15 min. After washing with PBS, the slices were incubated with a blocking solution (5% BSA in PBS) for 30 min. And then with the sections were incubated with anti-GRP78 (Zenbio, China; 1:100 dilution), anti-CHOP (Proteintech, China, 1:100 dilution), anti-P53 (Servicebio, China; 1:200 dilution), anti-P21 (Zenbio, China; 1:100 dilution), anti-P16 (cohesionbio, UK; 1:100 dilution), anti-RB(CST, USA; 1:300 dilution), anti-SLC43a3 (Novus, USA, 1:200 dilution) primary antibodies at 4 °C overnight. Finally, the sections were incubated with a secondary antibody for 1 h at room temperature and counterstained with hematoxylin.

For immunofluorescence (IF) staining, NP cells were seeded into 15-mm confocal dishes, fixed with 4% paraformaldehyde, permeabilized using Triton 0.1%, and blocked with goat serum. The anti-GRP78 (Zenbio, China; 1:100 dilution), anti-CHOP (Proteintech, China, 1:100 dilution), anti-P53 (Servicebio, China; 1:100 dilution), anti-P21 (Zenbio, China; 1:100 dilution), anti-P16 (Cohesionbio, UK; 1:100 dilution), anti-RB (CST, USA; 1:100 dilution) antibodies, anti-SLC43A3 (Santa, USA, 1:100 dilution) was incubated overnight at 4 °C. Alexa Fluor-594 or Alexa Fluor-488 was used as the secondary antibody. The samples were visualized by confocal microscopy (Zeiss, LSM 800, Germany).

### EdU staining

EdU staining was performed using a BeyoClick™ EdU-590 Kit (Beyotime, China). NP cells were cultured in confocal dishes, and 10 µM regent was added to the medium, which was incubated for 3 h at 37 °C to enable EdU labeling. The cells were fixed with 4% paraformaldehyde and permeabilized with 0.5% Triton X-100. Subsequently, the cells were incubated in the Click solution for 30 min and stained with DAPI. The samples were visualized by confocal microscopy (Zeiss, LSM 800, Germany).

### Senescence-associated β-galactosidase (SA-β-Gal) staining

SA-β-Gal staining was performed using an SA-β-Gal Staining Kit (Beyotime, China) according to the manufacturer’s protocol. Briefly, NP cells were fixed with a fixative solution and then incubated with fresh SA-β-Gal staining solution at 37 °C for 8 h. SA-β-Gal-positive senescent NP cells appeared blue under a light microscope.

### Transmission electron microscopy (TEM)

The samples were fixed at 4 °C in 2.5% glutaraldehyde overnight, treated with 1% osmium tetroxide for 2 h at 4 °C, and then dehydrated using a graded ethanol series (from 30%, 50%, 70%, 90% to 100% ethanol). After dehydration, the samples were treated with an embedding medium containing propylene oxide. Ultrathin sections were cut using an LKB-V ultramicrotome, stained with uranyl acetate and lead citrate, and finally visualized using a transmission electron microscope.

### Fatty acid uptake assay

BODIPY-PA is a fluorescently labeled palmitate analog. Palmitate uptake ability was determined by measuring the relative fluorescence intensity in cells after incubation with BODIPY-PA (Cayman, USA). Briefly, NP cells were cultured in confocal dishes and then incubated in 2.0 μM BODIPY-PA at 37 °C for 3 h. After washing, the samples were detected by confocal microscopy (Zeiss, LSM 800, Germany).

### Lipid droplet staining

Lipid droplets were stained with BODIPY 493/503 (Cayman, USA). Fixed cells were incubated for 30 min at 37 °C in the dark with 0.2 g/mL BODIPY 493/503 in solution. Then, the cells were washed and counterstained with DAPI. The samples were visualized using a confocal microscope (Zeiss, LSM 800, Germany). The quantification of lipid droplets per cell was performed for measurement.

Oil Red staining was performed using an oil red O staining kit (Beyotime, China) with modifications. NP cells were fixed with 4% paraformaldehyde for 10 min and then stained with oil red O solution for 15 min at room temperature.

### Animal experiments

All of the animal experiments were conducted following a protocol approved by the animal ethics committee. We have complied with all relevant ethical regulations for animal use. Three-month-old male Sprague‒Dawley rats were used for the animal experiments according to methods reported previously^4,45^. After anesthetization, a rat intervertebral disc (Co8/9) was punctured with a 20-gauge needle from the dorsal side, and the appropriate needle-insertion depth was predetermined to be approximately 5 mm. The needle was inserted into the disc along the vertical direction and then rotated in the axial direction by 180° and held for 10 s. To investigate the effect of PA on IDD and the potential protective potential of SLC43A3 in vivo, we prepared four solutions, PBS, PA, a mixture of PA and a control adenovirus, and a mixture of PA and SLC43A3-overexpressing adenovirus for intradiscal injection. Rat SLC43A3-overexpressing adenovirus and control adenovirus were constructed by Shanghai Genomeditech Company (Shanghai, China). Twenty rats were randomly assigned to four groups: the PBS+normal control group (NC, *n* = 5); the PA group, which consisted of IDD model rats injected of PA (200 µm, 3 µl); the PA+CTL AAV group, which consisted of IDD model rats injected with a mixture of PA+control adenovirus (1.2 × 10^10^ vector genomes in 3 µl); and the PA+SLC43A3 group, which consisted of IDD model rats injected with a mixture of PA+SLC43A3 adenovirus (1.2 × 10^10^ vector genomes in 3 µl). The injection procedure was performed immediately and 7 days post-IDD surgery. All rats were allowed free, unrestricted weight-bearing activity.

### Animal MRI evaluation

At eight weeks after the puncture surgery, all the rats were examined using MRI (GE, USA). Sagittal T2-weighted images in the median sagittal plane were obtained to evaluate the severity of intervertebral disc degeneration. The severity of IDD in the MR images was evaluated using the Pfirrmann grading system.

### Statistics and reproducibility

The data are presented as the mean and standard deviation (SD) from at least three repeats. Student’s *t*-test was used for statistical comparisons between the two groups. For multiple group comparisons, a one-way analysis of variance with Tukey’s post hoc test was used. At least three repetitions were used for each experiment. Statistical analyses were carried out using GraphPad Prism 8 (GraphPad Software, Inc.). *P* < 0.05 was considered statistically significant.

### Supplementary information

Supplementary Information

Description of Additional Supplementary Files

Supplementary Data

## Data Availability

Source data underlying main figures are presented in Supplementary data. Raw RNA-Seq data are available in NCBI Sequence Read Archiv under the following number PRJNA1100191. The proteomics data have been deposited to the ProteomeX change Consortium via the iProX partner repository with the dataset identifier PXD051351. The Uncropped blots are shown in Supplementary Fig. 2. All other data are available from the corresponding author upon reasonable request.
