# Supplementary material for: Palmitic acid induces lipid droplet accumulation and senescence in nucleus pulposus cells via ER-stress pathway
Source: Commun Biol. 2024 May 7;7:539. doi: 10.1038/s42003-024-06248-9 (PMC11076507; doi:10.1038/s42003-024-06248-9)

Palmitic acid induce lipid droplet accumulation and senescence in nucleus pulposus cells via ER-stress pathway

Xi Chen[1#], Kun Chen[1#], Jun Hu[1], Yijun Dong[1], Menglong Zheng[3], Jiang Jiang[1], Qingsong Hu[2*], Wenzhi Zhang[1*]

[1]Department of Orthopedics, The First Affiliated Hospital of USTC, Division of Life Sciences and Medicine, University of Science and Technology of China, Hefei, 230001, China.

[2]Department of Hepatobiliary Surgery, Anhui Province Key Laboratory of Hepatopancreatobiliary Surgery, The First Affiliated Hospital of USTC, Division of Life Sciences and Medicine, University of Science and Technology of China, Hefei 230001, China.

[3]Department of radiology, The First Affiliated Hospital of USTC, Division of Life Sciences and Medicine, University of Science and Technology of China, Hefei, 230001, China.

[#] These authors contributed equally to this work

***Corresponding Authors**

Qingsong Hu, E-mail: qshu@ustc.edu.cn;

Wenzhi Zhang, E-mail: wenzhizhang@ustc.edu.cn;

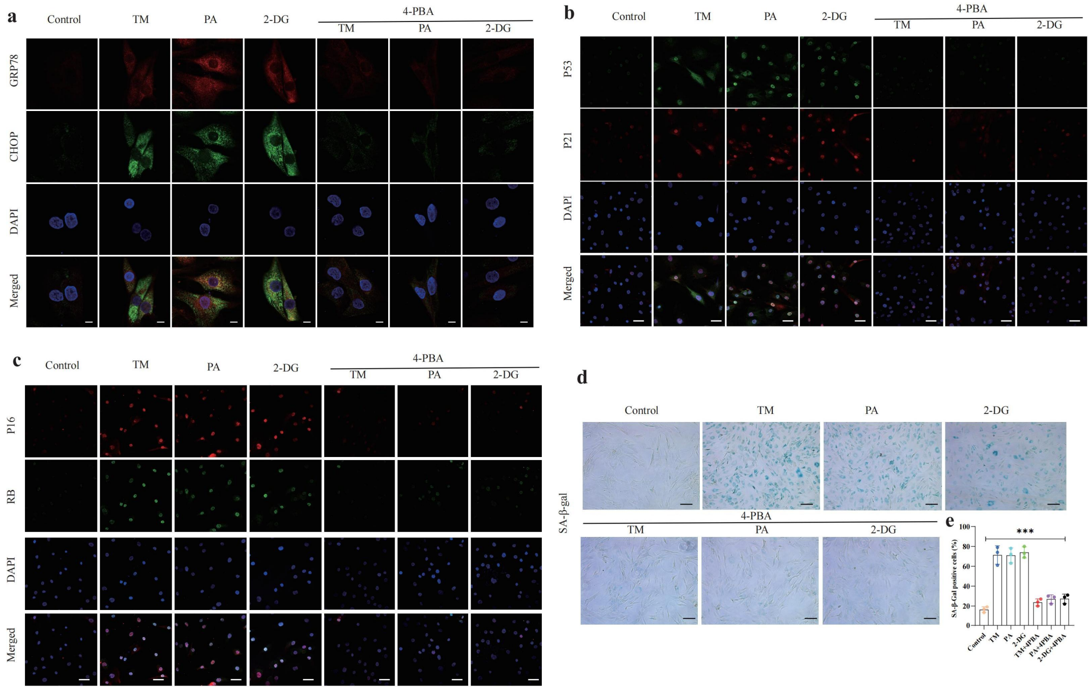

Supplementary figure 1. NP cell senescence caused by ER stress activation (a). ER-stress inducing agents tunicamycin (TM), palmitic acid (PA) or 2-deoxyglycose(2-DG) activate the expression of ER-stress makers GRP78 and CHOP, while ER-stress inhibitors block the express of GRP78 and CHOP; Scale bar:10μm. (b, c) Representative IF staining of senescent makers P53, P21, P16 and RB under ER-stress condition. Scale bar:50μm. (d, e). Representative SA-β-gal staining of human NP cells under the ER-stress condition. Scale bar:100μm. The positive SA-β-gal cells are shown in the statistical chart. n=3. ***P<0.001.

Supplementary figure 2. Uncropped and unedited Western blots of all the indicated figures in main figures.

Figure 4a

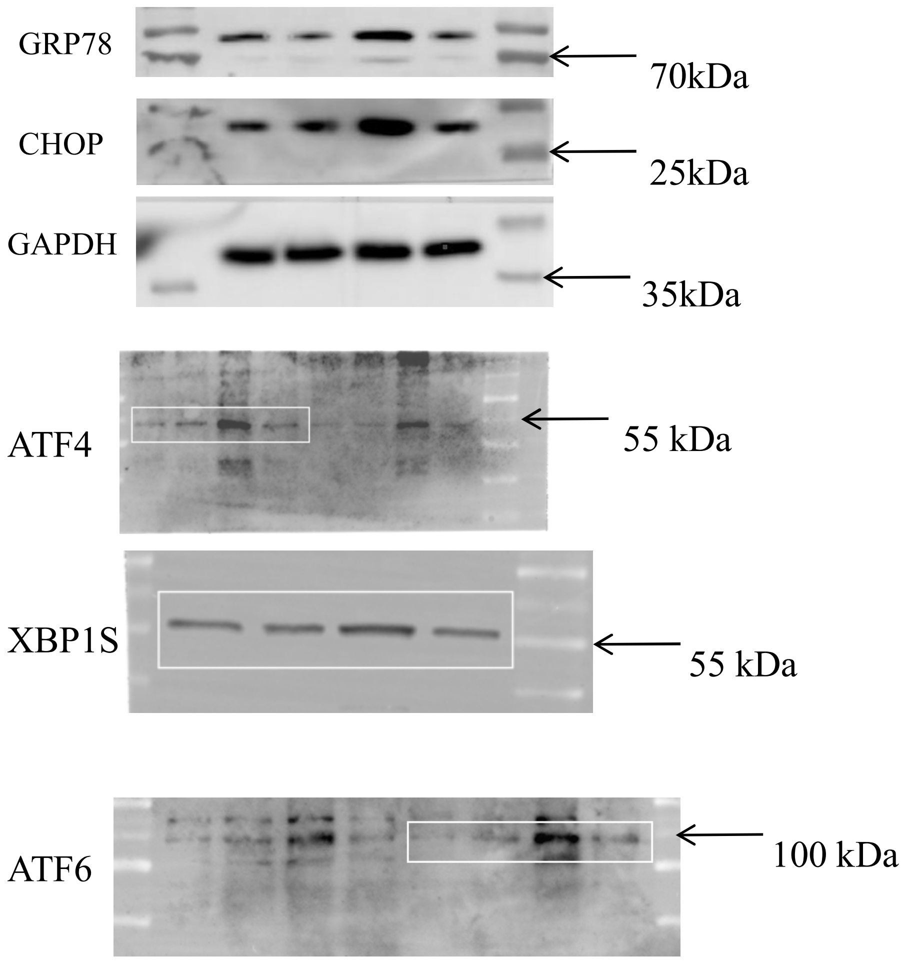

Figure 4h

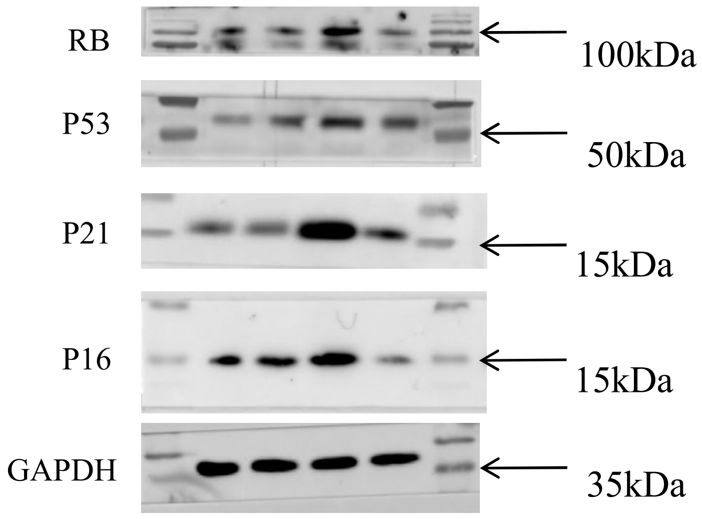

Figure 6a

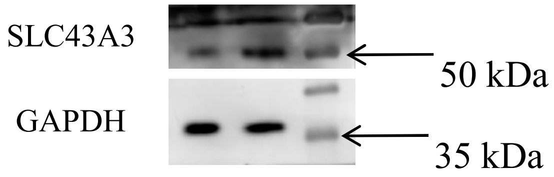

Figure 6e

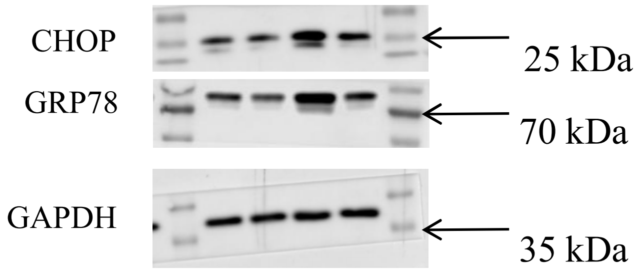

Supplement: Supplementary file 1 — Supplementary Information [file 42003_2024_6248_MOESM1_ESM.pdf]
