# Supplementary material for: Palmitic acid induces lipid droplet accumulation and senescence in nucleus pulposus cells via ER-stress pathway
Source: Commun Biol. 2024 May 7;7:539. doi: 10.1038/s42003-024-06248-9 (PMC11076507; doi:10.1038/s42003-024-06248-9)
Supplement: Supplementary file 2 — Description of Additional Supplementary Files [file 42003_2024_6248_MOESM2_ESM.pdf]

Description of Additional Supplementary Files

File name: Supplementary Data 1

Description: Source Data used to generate the main figures
